# Extensive mapping of an innate immune network with CRISPR

**DOI:** 10.15252/msb.20156373

**Published:** 2015-07-24

**Authors:** Michael Aregger, Traver Hart, Jason Moffat

**Affiliations:** 1 Donnelly Centre, University of TorontoToronto, ON, Canada; 2 Department of Molecular Genetics, University of TorontoToronto, ON, Canada

## Abstract

The application of the CRISPR-Cas9 system marks a major breakthrough for genetic screens, particularly in mammalian cells where high-throughput targeted gene editing has been lacking. Parnas *et al* (2015) apply this screening technology to mouse bone marrow-derived dendritic cells in order to study the regulation of the immune response triggered by PAMPs. Through integrated analysis of gene knockouts in conjunction with changes in protein and mRNA expression, CRISPR screens are facilitating dissection of immune regulatory networks at unprecedented resolution.

Genetic screens are powerful to identify individual genes or gene networks which contribute to a specific biological phenotype or disease context. The application of CRISPR-Cas9 genome editing technology to forward genetic screens in a diverse array of cell types is poised to fundamentally enhance the power of such genetic screens in mammalian cells compared to RNA interference, which until recently was the best available technology but suffers from incomplete gene knockdown and extensive off-target effects (Kaelin, 2012; Hart *et al*, 2014; Shalem *et al*, 2014; Wang *et al*, 2014). Over the last ∼year, genome-wide gene knockout screens using the CRISPR-Cas9 system have been performed in mammalian cancer and stem cell lines identifying genes essential for proliferation and uncovering genes mediating resistance to drugs and toxins (Koike-Yusa *et al*, 2014; Platt *et al*, 2014; Shalem *et al*, 2014; Wang *et al*, 2014; Zhou *et al*, 2014). Moving one step further, an *in vivo* genome-wide CRISPR screen has been reported in mice identifying loss-of-function mutations that drive tumour growth and metastasis (Chen *et al*, 2015). These initial negative and positive selection screens have established the feasibility of genome-scale CRISPR screens and have provided a glimpse of its power for yielding novel biological insight at high sensitivity and specificity.

Now this technology has been used to deconstruct a complex biological process, the first application of CRISPR-Cas9 to a systems-level biological question in mammalian cells. Parnas and colleagues report screening primary mouse bone marrow-derived dendritic cells (DCs), where they dissect the regulatory network associated with induction of tumour necrosis factor or TNF (Parnas *et al*, 2015). The authors use bone marrow-derived DCs isolated from Cas9-expressing transgenic mice to study the innate immune response to lipopolysaccharide (LPS) through Toll-like receptors (TLR) (Fig1). Parnas *et al* (2015) performed a genome-wide pooled CRISPR screen on these *ex vivo* cells and, after activation with LPS, sorted the cells based on high or low protein expression of the inflammatory cytokine TNF. The primary screen identified most known regulators of TNF expression and TLR4 signalling, as well as novel hits that were validated with individual single-guide RNAs (sgRNAs). This analysis was followed by a deeper secondary screen comprising the top ranked 2,569 genes and revealed additional regulators of TNF expression with greater sensitivity.

**Figure 1:**
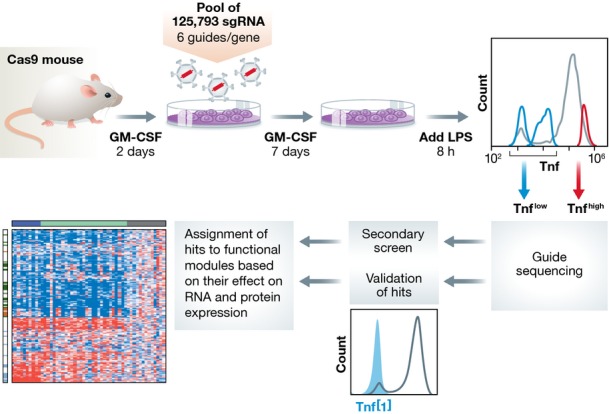
A genome-wide pooled CRISPR screen in mouse primary immune cells to dissect regulatory networks Design of the genome-wide primary and secondary screens by FACS sorting, deep sequencing and subsequent assignment of hits to functional modules based on their effect on RNA and protein expression to map immune regulatory networks [heatmap adapted from Parnas *et al* (2015), ©CellPress].

Subsequently, the authors assigned all the known and novel hits to functional modules based on their impact on RNA and protein expression of selected markers of dendritic cell function. Besides the known regulators of TLR signalling, two modules comprising genes that were not previously implicated in TNF regulation or inflammatory gene expression were identified including (i) the OST protein glycosylation complex and ER folding and translocation pathway and (ii) the PAF complex that is involved in the regulation of transcriptional elongation. Although it is not clear how the OST and PAF complexes impact the TLR pathway on a molecular level, this study demonstrates the need for unbiased exploration of functional networks in order to explore how biological functions are linked within a cell.

One of the novelties of the CRISPR screen performed by Parnas and colleagues is the application of the available technology to primary cells in order to study immune signalling related to infectious disease. A similar study has recently been reported which used a genome-wide RNA interference screen to uncover innate immune signalling triggered by pathogen-associated molecular patterns (PAMPs) (Gaudet *et al*, 2015). The authors of that study report a novel PAMP and detect a number of components of the TRAF complex, but the screen was far from saturating. The CRISPR-Cas9 technology may hold the potential to perform saturating genetic screens in mammalian cells, enabling the decryption of near-complete molecular pathways. Parnas and colleagues applied the combination approach of a CRISPR screen with cell sorting in order to reveal regulators of particular proteins or biological phenotypes beyond cellular proliferation. This approach could be applied to a wide range of targets and bears great potential to identify uncharacterized regulatory networks.

Despite the fast uptake of the CRISPR-Cas9 system, the potential pitfalls of this technology should not be forgotten as we learn to apply this incredible technology in genetic screens: for example, the detection of false-positive and false-negative hits due to low library coverage or off-target effects should be considered when performing genome-wide screens. The authors effectively addressed these issues by evaluating screen performance against appropriate positive controls, for example gene ontology annotations and a reference set of essential and nonessential genes (Hart *et al*, 2014), and by measuring the validation frequency of hits in the primary screen. Parnas and colleagues performed a deeper secondary screen in order to reduce false negatives due to limited cell number compared to the size of the genome-wide library. Even with these pitfalls in mind, genome-wide CRISPR screens have begun to revolutionize the study of regulatory networks and open the path to novel discoveries at the systems level.

## References

[b1] Chen S, Sanjana NE, Zheng K, Shalem O, Lee K, Shi X, Scott DA, Song J, Pan JQ, Weissleder R, Lee H, Zhang F, Sharp PA (2015). Genome-wide CRISPR screen in a mouse model of tumor growth and metastasis. Cell.

[b2] Gaudet RG, Sintsova A, Buckwalter CM, Leung N, Cochrane A, Li J, Cox AD, Moffat J, Gray-Owen SD (2015). Cytosolic detection of the bacterial metabolite HBP activates TIFA-dependent innate immunity. Science.

[b3] Hart T, Brown KR, Sircoulomb F, Rottapel R, Moffat J (2014). Measuring error rates in genomic perturbation screens: gold standards for human functional genomics. Mol Syst Biol.

[b4] Kaelin WG (2012). Use and abuse of RNAi to study mammalian gene function. Science.

[b5] Koike-Yusa H, Li Y, Tan E-P, Velasco-Herrera MDC, Yusa K (2014). Genome-wide recessive genetic screening in mammalian cells with a lentiviral CRISPR-guide RNA library. Nat Biotechnol.

[b6] Parnas O, Jovanovic M, Eisenhaure TM, Herbst RH, Dixit A, Ye CJ, Przybylski D, Platt RJ, Tirosh I, Sanjana NE, Shalem O, Satija R, Raychowdhury R, Mertins P, Carr SA, Zhang F, Hacohen N, Regev A (2015). A genome-wide CRISPR screen in primary immune cells to dissect regulatory networks. Cell.

[b7] Platt RJ, Chen S, Zhou Y, Yim MJ, Swiech L, Kempton HR, Dahlman JE, Parnas O, Eisenhaure TM, Jovanovic M, Graham DB, Jhunjhunwala S, Heidenreich M, Xavier RJ, Langer R, Anderson DG, Hacohen N, Regev A, Feng G, Sharp PA (2014). CRISPR-Cas9 knockin mice for genome editing and cancer modeling. Cell.

[b8] Shalem O, Sanjana NE, Hartenian E, Shi X, Scott DA, Mikkelsen TS, Heckl D, Ebert BL, Root DE, Doench JG, Zhang F (2014). Genome-scale CRISPR-Cas9 knockout screening in human cells. Science.

[b9] Wang T, Wei JJ, Sabatini DM, Lander ES (2014). Genetic screens in human cells using the CRISPR-Cas9 system. Science.

[b10] Zhou Y, Zhu S, Cai C, Yuan P, Li C, Huang Y, Wei W (2014). High-throughput screening of a CRISPR/Cas9 library for functional genomics in human cells. Nature.

